# Defining the Construct of Health Behavior Maintenance

**DOI:** 10.1093/geroni/igaa057.3021

**Published:** 2020-12-16

**Authors:** Mina Raj, Janet Bettger, Susan Hughes, Jaime Hughes

**Affiliations:** 1 University of Michigan, Ann Arbor, Michigan, United States; 2 Duke University School of Medicine, Durham, North Carolina, United States; 3 University of Illinois at Chicago, Chicago, Illinois, United States

## Abstract

Prior research on health behavior maintenance has proposed several key constructs that distinguish this concept from initiation. Initiation is thought to depend upon action self-efficacy, goal setting, and outcome expectations. Maintenance, on the other hand, depends upon self-regulation, relapse prevention, recovery self-efficacy, and satisfaction with original outcome expectations. Although much prior research has focused on cognitive components of maintenance, there has been little attention to how higher-order cognitive processing and decision-making may be challenging for some older adults. This presentation will discuss a proposed conceptual model of health behavior maintenance, specifically as this construct applies to older adults. Special consideration will be given to how both normative and non-normative age-related changes (e.g., physical, cognitive, psychosocial) impact maintenance and how such changes might influence older adults’ goals and outcome expectations. Finally, individual-level maintenance will be discussed within a larger context of program sustainability at community and population levels.

